# Inactivation of p53 Rescues the Maintenance of High Risk HPV DNA Genomes Deficient in Expression of E6

**DOI:** 10.1371/journal.ppat.1003717

**Published:** 2013-10-24

**Authors:** Laurel D. Lorenz, Jessenia Rivera Cardona, Paul F. Lambert

**Affiliations:** McArdle Laboratory for Cancer Research, University of Wisconsin Madison School of Medicine and Public Health, Madison, Wisconsin, United States of America; Indiana University, United States of America

## Abstract

The human papillomavirus DNA genome undergoes three distinct stages of replication: establishment, maintenance and amplification. We show that the HPV16 E6 protein is required for the maintenance of the HPV16 DNA genome as an extrachromosomal, nuclear plasmid in its natural host cell, the human keratinocyte. Based upon mutational analyses, inactivation of p53 by E6, but not necessarily E6-mediated degradation of p53, was found to correlate with the ability of E6 to support maintenance of the HPV16 genome as a nuclear plasmid. Inactivation of p53 with dominant negative p53 rescued the ability of HPV16 E6STOP and E6SAT mutant genomes to replicate as extrachromosomal genomes, though not to the same degree as observed for the HPV16 E6 wild-type (WT) genome. Inactivation of p53 also rescued the ability of HPV18 and HPV31 E6-deficient genomes to be maintained at copy numbers comparable to that of HPV18 and HPV31 E6WT genomes at early passages, though upon further passaging copy numbers for the HPV18 and 31 E6-deficient genomes lessened compared to that of the WT genomes. We conclude that inactivation of p53 is necessary for maintenance of HPV16 and for HPV18 and 31 to replicate at WT copy number, but that additional functions of E6 independent of inactivating p53 must also contribute to the maintenance of these genomes. Together these results suggest that re-activation of p53 may be a possible means for eradicating extrachromosomal HPV16, 18 or 31 genomes in the context of persistent infections.

## Author Summary

Human papillomaviruses (HPVs) infect epithelial tissues. HPVs that infect mucosal epithelia cause infectious lesions in the anogenital tract and oral cavity. HPV infections are normally cleared by the immune system; however, in rare cases, infections can persist for years. Persistent infections by certain HPVs place one at a high risk of developing carcinomas of the cervix, other anogenital tissues, and the head/neck region. These HPVs are responsible for over 5% of all human cancers. For an HPV infection to persist, the viral circular genome must be maintained, i.e. replicated and inherited during cell division. In this study we define the mechanism by which the viral gene *E6* contributes to the maintenance of the HPV genome. We demonstrate that *E6* must inactivate the cellular factor, p53, for the viral genome to be maintained. Significantly, p53, is inactivated in many types of human cancers and because much research has been done on p53, promising new drugs have been identified that can re-activate p53. If such drugs can re-activate the p53 that has been inactivated by *E6*, then we hypothesize that these drugs could be used to cure patients with persistent HPV infections and thereby reduce their risk of developing HPV associated cancers.

## Introduction

Human papilloma viruses (HPVs) are small, non-enveloped icosahedral viruses that infect epithelial linings of the body and are the causative agents of warts. Infection is thought to arise when a virus particle enters a dividing basal epithelial cell, accessed through a wound in the epithelia, wherein its viral genome is delivered to the nucleus and viral genes begin to be expressed [1]. The papillomavirus life cycle is intricately tied to the differentiation of the stratified squamous epithelia that they infect, with progeny virus exclusively generated in the suprabasal compartment [2]–[5]. HPVs are classified as cutaneous or mucosotropic depending upon the type of epithelia they infect. A subset of the mucosotropic HPVs, the so-called high risk HPVs, including HPV genotypes 16, 18 and 31, are associated with approximately 5% of human cancers including the vast majority of cervical cancers as well as other anogenital cancers and a growing fraction of head and neck cancers [6]–[10]. An important requirement for the onset of HPV-associated cancers is persistent infection by these high risk HPVs [11]. Prophylactic HPV vaccines hold great promise in preventing new infections but do not eliminate pre-existing infections [12]. Developing the means to eliminate persistent high risk HPV infections would be of great value in reducing the risk of cancer among patients already infected with high risk HPVs.

The papillomavirus genome is an 8 kB circular double-stranded DNA that replicates as a nuclear plasmid in three distinct phases referred to as the establishment, maintenance and productive phases [13]. Establishment refers to the replication process by which the HPV genome establishes itself as a multi-copy extrachromosomal replicon, or nuclear plasmid, in undifferentiated basal cells. This stage of genome replication can be studied *in vitro* using short-term replication assays [14]–[16]. Using plasmids carrying the minimal *cis* element required for papillomavirus DNA replication, i.e. the viral origin of replication (*ori*), it has been demonstrated that two papillomaviral genes, *E1* and *E2*, are required for the establishment phase for multiple papillomaviruses including bovine papillomavirus type 1 (BPV1) as well as HPV6b, 11, 16 and 18 [15]–[17]. When the initial host cell harboring the established HPV genome undergoes cell division, the HPV genome is replicated and partitioned to daughter cells at a constant copy number of 50–200 extrachromosomal genomes per cell: this phase of HPV replication is referred to as maintenance [13], [18]–[20]. The expression of HPV genes required for maintenance differs among HPV types. While *E6* is required for maintenance of HPV11, 16 and 31 [21]–[23], expression of *E7* is only required for maintenance of HPV11 and 31, but is dispensable for maintenance of HPV16 and 18 [21]–[24]. Likewise, *E1∧E4* is important for maintenance of HPV16, but is dispensable for maintenance of HPV11, 18 and 31 [25]–[28]. Notably, while *E1* is required for establishment of HPV16, expression of *E1* has been shown recently to be dispensable for maintenance of HPV16 [29] as had been demonstrated previously for BPV1 [30]. Finally, during the productive phase of replication, the HPV genome is amplified to a high copy number per cell and this stage of replication is restricted to fully differentiated cells [18]. Here *E7* plays a critical role in amplification of HPV16 and HPV18 [22], [24] and *E6* is essential for robust amplification of HPV18 [31], [32]. Additional viral genes including *E1*∧*E4* and *E5* have been shown to contribute quantitatively to this phase of viral DNA replication of HPV16 and HPV31 [26], [27], [33].

Identifying viral and cellular genes of importance to different stages of the HPV replicative life cycle may help define new strategies for treating persistent HPV-infections. In this study, we sought to identify the roles of E6 that are necessary and sufficient for high risk HPV maintenance in the context of the entire HPV genome. The HPV E6 protein consists of approximately 150 amino acids and contains two zinc finger motifs. High risk HPV E6 proteins also contain a PDZ binding domain at the C terminus [34]–[41]. E6 is known to interact with a number of cellular proteins and modulate multiple cellular processes including apoptosis, transcription, interferon responses and immortalization [42]. The most well-known biochemical property of high risk E6 proteins is their ability to bind the ubiquitin ligase E6AP and the tumor suppressor p53 in a tripartite complex that drives proteasome-dependent degradation of p53 [43]–[45]. Because activation of p53 can lead to apoptosis or growth arrest, it has been hypothesized that E6 plays an important role in the prevention of either cellular process through its destabilization of p53, thereby allowing for the continued growth and expansion of cells harboring the HPV genome.

Previous studies of E6 mutants within the context of the HPV31 genome indicated that the ability of 31E6 to degrade p53 is required for the maintenance of HPV31 [23]. Specifically, an HPV31 genome carrying a three amino acid mutation F45Y/F47Y/D49H in E6 (E6 YYH), which in 16E6 had been previously shown to compromise E6-dependent degradation of p53 [46], was defective for maintenance of the HPV31 genome [23], [46]. Likewise, mutational studies of 16E6 placed within the context of the HPV31 genome demonstrated that 16E6 mutants deficient for decreasing p53 steady state levels are defective in the maintenance of the hybrid genome [47]. Thus, we hypothesized that in order for HPV16 to be maintained as an extrachromosomal genome, E6 must destabilize p53. This hypothesis is necessary to test within the context of the HPV16 genome for two reasons. First, the role of another HPV gene, *E7*, differs in its requirement for maintenance between different high-risk HPV types [22]–[24]. Secondly, the HPV31 E6 mutants used in previous studies were based off of mutants characterized from HPV16 E6, but HPV16 E6 mutants do not always behave the same way when introduced into a different HPV type [48], [49].

To test our hypothesis, we analyzed the capacity of the HPV16 genome carrying various mutations in the *E6* gene to be maintained as an extrachromosomal nuclear plasmid in normal immortalized human keratinocytes (NIKS), cells that retain wild type p53 [50]. The use of immortalized keratinocytes enabled us to directly examine the role of E6 that contributes to maintenance of the HPV16 genome independently of the role of E6 necessary for immortalization. Our mutational studies demonstrated that the ability of HPV16 E6 mutant genomes to inactivate p53, but not mediate p53 degradation, correlates with the ability of the viral genome to be maintained as an extrachromosomal nuclear plasmid. However, these and prior results using subtle mutations in E6 must be interpreted conservatively, as such mutants of E6 proteins have only been analyzed for a small subset of biochemical activities. To more clearly determine that inactivation of p53 is necessary and/or sufficient to account for the role of E6 in plasmid maintenance of high risk HPV16, 18 and 31, we performed complementation studies using cells expressing a dominant negative form of p53 (p53DD). We found that inactivation of p53WT (wild-type) complements the ability of HPV 16 E6-deficient genomes to be maintained at early passages. Surprisingly, we found that HPV31 E6-deficient genomes could be maintained at low levels in NIKS at early passages and HPV18 E6-deficient genomes could be maintained at low levels in NIKS and primary human foreskin keratinocytes. Inactivation of p53 also complemented the ability of HPV 18 and 31 E6 null genomes to be maintained as a nuclear plasmid at copy numbers similar to WT HPV genomes in NIKS, but did not rescue the ability of these genomes to be maintained at WT levels over several passages. These results demonstrate, for the first time, that inactivation of p53 is necessary for maintenance of HPV16, 18 and 31 genomes at WT levels, but additional functions of E6 may contribute to maintenance of these genomes.

## Methods

### Plasmids

Full-length clones of HPV16, HPV18 and HPV31 were subjected to site directed mutagenesis to generate mutations within the E6 open reading frame (ORF) as detailed in text S1. All mutant genomes were sequenced in their entirety to confirm the introduction of the desired mutation and the absence of spurious changes in the viral DNA sequence. See text S1 for further description of the plasmids used in this study and their sources.

### Cells

NIKS (Normal Immortalized KeratinocyteS) and primary human foreskin keratinocytes (HFKs) have been previously described by and were obtained from Lynn Allen-Hoffman [50]. Using previously described conditions for their culture in monolayer [51], NIKS and HFKs were transfected with recombinant HPV DNA genomes that had been released from their bacterial vector and re-circularized using T4 DNA ligase together with a plasmid conferring drug resistance (either resistance to G418 or blasticidin), and subjected to drug selection. Colonies that outgrew were pooled and expanded. This initial population of pooled colonies is referred to as passage 0 (P0). These populations of cells were serially passaged and cryopreserved for later studies. In some cases colony-derived, clonal populations were isolated and characterized as indicated in the text. Details on the culturing conditions, preparation of DNA for transfection, and DNA transfections are provided within text S1. To generate NIKS expressing p53DD and vector control transduced cells, cells were infected with pLXSNp53DD recombinant retrovirus that expresses the dominant negative form of p53, p53DD, or the control vector pLXSN retrovirus. Transductants were selected by growth of cells in the presence of G418, individual colonies were cloned, and expanded clones were subjected to p53-specific western analysis to identify clonal populations expressing p53DD. Further details regarding these steps are provided in text S1.

### Detection of HPV genomic DNA in NIKS

Total genomic or low molecular weight DNA was isolated from cells and subjected to HPV-specific Southern analysis as detailed in text S1. Briefly, DNA from equivalent numbers of cells were digested with indicated restriction enzymes overnight, electrophoresed on agarose gels, transferred to nylon membranes, and HPV DNA was detected by hybridization to pools of radioactively labeled, HPV genotype-specific, single-stranded oligonucleotides. Alternatively, HPV16 DNA was quantified by real-time quantitative PCR (qPCR) as detailed in text S1.

### Response of cells to actinomycin D

To assess the function of p53 (Entrez Gene ID: 7157) in cells, we monitored the responses of cells to actinomycin D. Briefly, cells treated with 0.5 nM–5 nM actinomycin D (Sigma) or vehicle (dimethyl sulfoxide (DMSO) (Sigma)) for 24 hours were harvested, fixed in ice cold ethanol, stained with propidium iodide, subjected to flow cytometry using a Becton Dickenson FACSCalibur, and cell cycle profiles analyzed using Flow Jo Version 9.4.11 software. The G1/S ratio was calculated by dividing the percentage of cells in G1 by the percentage of cells in S phase. The magnitude change in the G1/S ratio of control NIKS after vehicle and actinomycin D treatment was compared to the magnitude change in the G1/S ratio of experimental NIKS after vehicle and actinomycin D treatment using the Sen-Adichie test for parallelism in MSTAT version 5.5.1 software. As another means of assessing p53 function, steady state levels of p21 (Entrez Gene ID: 1026) were determined by western blot analysis of cells likewise treated with actinomycin D or vehicle. Further details on these actinomycin D-based experiments are provided in text S1.

## Results

### E6 is required for establishment and/or maintenance of HPV16

**Figure 1:**
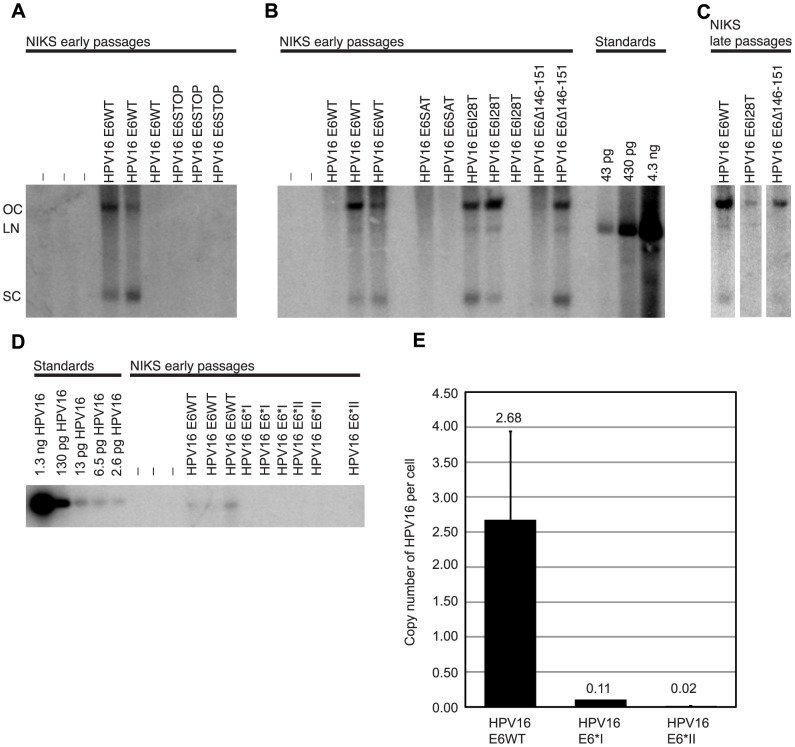
HPV16 E6 is necessary for the stable maintenance of HPV16 as an extrachromosomal genome. Shown are HPV16-specific Southern analyses of total genomic DNA isolated from early (A and B, passage 2) or late (C, passage 6 or higher) NIKS transfected with the indicated wild-type or E6 mutant HPV16 genomes (lanes labeled with the symbol “-” were transfected with the drug selection plasmid but not HPV16 DNA – see methods). For each sample, 20 µg of total genomic DNA was digested with XhoI (a noncutter of HPV16) prior to Southern analysis. Indicated at the left is the position of migration of open circular (OC), linear (LN) and supercoiled circular (SC) forms of HPV16 DNA. At the right side of panel B are copy number standards: 1.7 copies of HPV/cell from 20 µg DNA is equal to 43 pg of HPV. (A) HPV16 E6WT genomes replicated extrachromosomally as evidenced by the presence of open circular and supercoiled circular forms of the viral genome, whereas HPV16 E6STOP genomes were not detected. (B) HPV16 E6SAT was not maintained extrachromosomally as evidenced by the lack of open circular or supercoiled circular forms of HPV16 DNA. However, the HPV16 E6I128T and E6Δ146–151 mutant genomes replicated extrachromosomally with copy numbers comparable to the wild-type genome. In Southerns not shown, BamHI/DpnI digestions revealed that all of the detectable genomes were DpnI resistant. In (C) can be observed the continued maintenance of the HPV16 E6WT, E6I128T and E6Δ146–151 genomes as extrachromosomal nuclear plasmids in later passages of NIKS (passages 6 or higher). In (D) low molecular weight DNA (1.5*10^6^ cells worth) was digested with BamHI/DpnI. Unit length forms of HPV16 E6WT but not HPV16 E6*I and E6*II genomes could be detected. qPCR in (E) confirmed that the HPV16 E6WT genome was present at approximately 2.5 copies/cell while HPV16 E6*I and E6*II were present at .11 and 0.02 copies/cell respectively. Error bars represent the standard deviation.

To determine the requirements of E6 in the establishment and maintenance of the HPV16 genome, we introduced various mutations within the E6 gene in the context of the full-length wild type HPV16 genome. To determine if E6 was required for establishment and/or maintenance of the HPV16 genome as a nuclear plasmid, a stop codon at amino acid 7 was introduced by mutating nucleotide 122 of HPV16 from G to T to create the HPV16 E6STOP mutant genome. The HPV16 E6WT and HPV16 E6STOP genomes were excised from their bacterial vector, re-circularized and each was transfected with a plasmid expressing an antibiotic resistance gene into NIKS. Drug resistant colonies arising 2–3 weeks after selection were pooled to generate a population of cells (referred to as passage 0) and were further passaged. Total genomic DNA was harvested from the expanded populations of cells at early passages (passage 1 and 2) and analyzed by Southern hybridization using an oligonucleotide probe set specific for HPV16. To determine the presence of mammalian replicated, extrachromosomal HPV16 genomes, total genomic DNA was digested overnight with BamHI plus DpnI or XhoI alone. Using these restriction enzyme digestions, we were able to: 1) discriminate HPV16 genomes that had replicated in the human cells from input transfected DNA because the latter is selectively sensitive to DpnI digestion, 2) determine if the viral genome was being maintained as an extrachromosomal plasmid by looking for the presence of circular viral DNA genomes in the samples cut with XhoI (non-cutter of HPV16), 3) determine if the viral DNA is integrated by looking for non-unit length viral DNA fragments in the samples cut with BamHI (single cutter of HPV16 - data not shown), and 4) estimate copy number of the viral genome. The HPV16 E6WT genome was capable of replicating extrachromosomally in 56% of populations analyzed, but the HPV16 E6STOP genome was deficient in its ability to replicate extrachromosomally based upon the absence of detectable circular viral genomes (Figure 1A and Table 1). These results demonstrate that E6 is required for establishment and/or maintenance of HPV16.

**Table 1:** Frequency of NIKS populations harboring extrachromosomal HPV16 DNA.

Transfected viral DNA	Fraction of populations analyzed that were positive for extrachromosomal HPV16 (%)
None	0/5 (0%)
HPV16 E6WT	5/9 (56%)
HPV16 E6STOP	0/7 (0%)
HPV16 E6SAT	0/6 (0%)
HPV16 E6I128T	6/9 (67%)
HPV16 E6Δ146–151	4/7 (57%)

Since the HPV16 E6STOP genome contains a stop codon at amino acid 7, this should also inhibit expression of E6 splice products, E6*I and E6*II [35], [36]. To determine if one of these proteins was sufficient to support maintenance of HPV16 genomes, NIKS were transfected with HPV16 E6*I and HPV16 E6*II mutant genomes which express 16E6*I or 16E6*II but not full length 16E6. Southern hybridization and qPCR analysis of NIKS populations transfected and selected for with these mutant HPV16 genomes revealed that expression of E6*I or E6*II in the absence of full-length E6 did not rescue efficient replication of the viral genome. The low copy number levels for the HPV16 E6*I and HPV16 E6*II mutant genomes were sufficiently low that it was undetectable by Southern hybridization (Figure 1D). When quantified by qPCR, the copy numbers of the HPV16 E6*I and E6*II mutant viral genomes were 0.11 and 0.02 copies per cell copy, respectively, which was well below the 2.68 copies per cell observed for HPV16 E6WT genome (Figure 1E).

### Reduced steady state levels of p53 are not required for stable maintenance of HPV16

After determining that E6 was required for stable maintenance of the HPV16 genome, we were interested in identifying the activities of 16E6 that are necessary for stable maintenance of HPV16. To this end, we examined the maintenance of HPV16 genomes containing E6 mutants that differed in their ability to reduce p53 steady state levels. Compared to 16E6WT, the 16E6 R8S/P9A/R10T (E6SAT) mutant is deficient for binding p53, decreasing p53 steady state levels, inhibiting p53-dependent transactivation and attenuating a p53 dependent G1/S growth arrest [39], [52]–[55]. While deficient for inhibiting p53 activity, the 16E6SAT mutant still binds E6AP and increases telomerase activity at levels comparable to 16E6WT [39], [53], [54]. The 16E6I128T mutant binds both E6AP and p53 at 1–5% of the level observed with E6WT and is deficient for decreasing p53 steady state levels, but can prevent E7 induced acetylation of p53 at lysine 382 [48], [56]. Southern analysis of NIKS transfected with these mutant genomes indicated that the HPV16 E6SAT genome was deficient for plasmid maintenance in NIKS, but the HPV16 E6I128T genome was competent for being maintained as an extrachromosomal genome in NIKS (Figure 1B and Table 1). Populations of NIKS harboring the HPV16 E6I128T extrachromosomal genome were further passaged to determine if the genome could be stably maintained. We found that the HPV16 E6I128T genome could be maintained extrachromosomally over at least 8 passages (Figure 1C).

### The PDZ binding domain of E6 is not required for stable maintenance of HPV16

We were also interested in determining if the C terminus of E6, which is involved in binding PDZ domain containing proteins [38], [39], is required for maintenance of HPV16. To test this, we used the HPV16 E6Δ146–151 mutant genome in which nucleotides 539–556 were deleted. While lacking the PDZ binding domain of E6, 16E6Δ146–151 is still able to induce telomerase activity, bind p53 at 33% of the levels of 16E6WT and mediate degradation of p53 at 67% of the levels of 16E6WT [54], [55]. When transfected into NIKS, this mutant genome was stably maintained as a nuclear plasmid over 8 passages (Figures 1B, 1C and Table 1). This result demonstrates that the PDZ binding domain of 16E6 is not required for stable maintenance of HPV16.

### The ability of 16E6 mutant genomes to be maintained correlates with inactivation of p53

**Figure 2:**
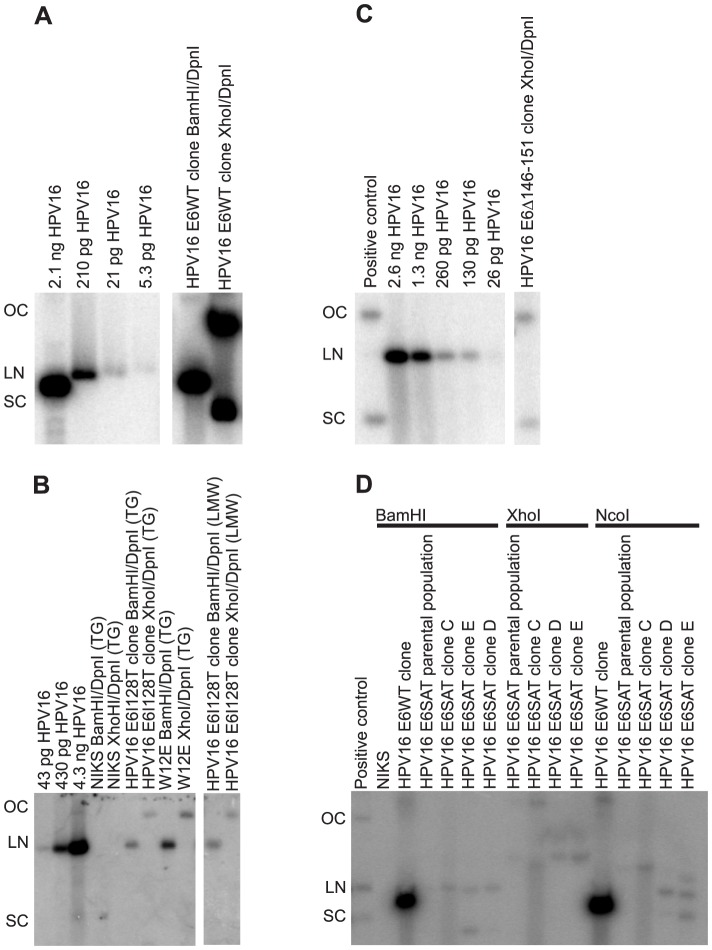
HPV16 E6WT, E6I128T and E6Δ146–151 mutant genomes but not HPV16 E6SAT are maintained extrachromosomally in clones. (A) HPV16 WT replicated extrachromosomally in a clone as indicated by the presence of open circular (OC), linear (LN) and supercoiled (SC) HPV16 DNA after XhoI/DpnI digestion of total genomic DNA. Passage 6 DNA is shown. (B) The HPV16 E6I128T clone replicated extrachromosomally as indicated by the presence of open circular DNA after XhoI/DpnI digestion of total genomic DNA (TG) and low molecular weight DNA (LMW). Digestion of this DNA with BamHI/DpnI indicates the presence of only unit length forms of HPV16 E6I128T. Note that the positive control, total genomic DNA from W12e cells (which harbor extrachromosomal HPV16) is also present at just open circular form after XhoI/DpnI digestion. Passage 6 DNA is shown. (C) The HPV16 E6Δ146–151 clone is also extrachromosomal as determined by the presence of open circular, linear and supercoiled DNA after XhoI/DpnI digestion of low molecular weight DNA. Passage 6 DNA is shown. (D) Low molecular weight DNA from four clones of NIKS harboring HPV16 E6SAT genomes was digested with indicated restriction enzymes. After XhoI digestion, none of the clones contained DNA at open circular, linear or extrachromosomal sizes. This demonstrates that all HPV16 E6SAT clones have integrated HPV16. The HPV16 E6SAT clone C also has two different DNA banding patterns after digestion with two separate single HPV16 cutters (BamHI and NcoI). This further demonstrates that HPV16 E6SAT is integrated in clone C. Lanes marked positive control in (C) and (D) are 7.9 kB circular plasmids containing segments of the HPV16 genome.

**Figure 3:**
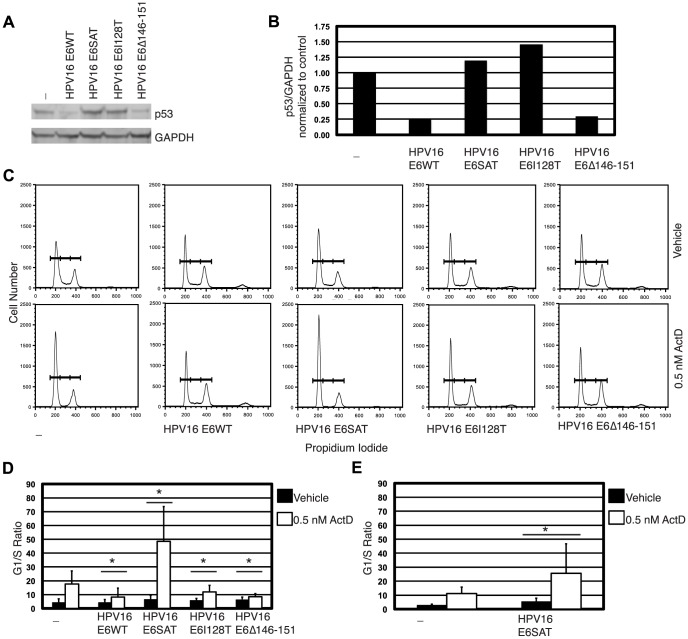
The ability of E6 mutant genomes to be stably maintained correlates with inactivation of p53. Shown in panel (A) is a p53-specific western blot analysis of clones of NIKS that harbored wild type or E6 mutant HPV16 genomes. GAPDH was used as a loading control. Compared to HPV negative NIKS (-), p53 steady levels were reduced in NIKS harboring HPV16 E6WT and E6Δ146–151 mutant genomes but not a clone of NIKS harboring HPV16 E6SAT (clone C) or a clone harboring the HPV16 E6I128T genome. Panel (B) is a bar graph of the densitometry in the p53/GAPDH ratio shown in (A) normalized to control (HPV negative NIKS (-). Shown in panel (C) are flow cytometric profiles of propidium iodide-dependent fluorescence of the same clonal cells populations characterized in panel A that had been treated for 24 hours with 0.5 nM actinomycin D (ActD, bottom) or vehicle (top). Shown in panel (D) is the G1/S ratios (percent of cells in G1 phase of the cell cycle divided by the percent of cells in the S phase) for the data presented in panel B. Panel (E) indicates the average actinomycin D induced G1/S ratio in three HPV16 E6SAT clones (clone C,D and E). The Sen-Adichie test for parallelism was used to compare the magnitude of change in the G1/S ratio between vehicle and actinomycin D treatment for each clone and this magnitude of change in the G1/S ratio was compared to NIKS not harboring HPV16 (-). An asterisk indicates p values less than 0.002.

To ascertain if p53 was functional in NIKS harboring the viral genomes as extrachromosomal, nuclear plasmids (HPV16 E6WT, E6I128T and E6Δ146–151) or in NIKS harboring viral genomes in the integrated state (HPV16 E6SAT), previously frozen populations of NIKS transfected with these genomes were thawed and individual colonies were isolated and expanded. Southern blot analysis confirmed the genomic status of the HPV16 mutant genomes in these clonal populations (Figure 2 A–D). As with the original populations, the derived clone harboring either the HPV16 E6I128T or E6Δ146–151 genomes retained the respective genome as a nuclear plasmid over at least 6 passages (Figure 2 B–C). Consistent with results from our analysis of populations, the HPV16 E6SAT genome was found to be integrated in all of the three screened clones (Figure 2D). The steady state level of p53 in each of these clones was analyzed by western blot. As predicted, the clone harboring the HPV16 E6WT or E6Δ146–151 genome had reduced steady state levels of p53 compared to non-transfected NIKS while NIKS harboring the HPV16 E6SAT or E6I128T genome did not display decreased steady state levels of p53 compared to non-transfected NIKS (Figure 3A). We also analyzed the steady state levels of p53 in at least four independent populations of NIKS harboring HPV16 E6WT or E6I128T mutant genomes and saw similar results (results not shown). Thus, the capacity of the HPV16 genome to be maintained as an extrachromosomal, nuclear plasmid does not correlate with the ability of E6 to reduce the steady state levels of p53; specifically, the E6I128T mutant HPV16 genome, which stably replicates extrachromosomally, fails to cause a decrease in p53 protein levels in the cells.

Given this result, we wanted to determine the functional status of p53 in these HPV-positive epithelial cells. p53 function can be tested by measuring the response of cells to actinomycin D. Actinomycin D induces a p53-dependent G1 growth arrest and HPV16 E6 can inhibit this actinomycin D-induced growth arrest [55], [57]–[59]. A clone of HPV-negative NIKS or a clone of NIKS harboring the HPV16 E6 WT or E6 mutant genomes was treated with vehicle (DMSO) or 0.5 nM actinomycin D for 24 hours, fixed, stained with propidium iodide and analyzed by flow cytometry to determine the percentage of cells in G1, S and G2/M phases of the cell cycle (Figures 3C and 3D). In the presence of actinomycin D, a higher percentage of NIKS accumulated at G1 and fewer cells were found in S phase resulting in an increased G1/S ratio compared to vehicle (Figure 3D). Using the Sen-Adichie test for parallelism, the magnitude of change in the G1/S ratio of HPV negative NIKS after vehicle and actinomycin D treatment was compared to NIKS harboring each of the different HPV16 E6WT or mutant genomes. NIKS harboring HPV16 E6WT, E6I128T and E6Δ145–151 genomes significantly reduced the magnitude of change in the G1/S ratio after actinomycin D treatment compared to NIKS not containing HPV (p<0.002, Figure 3D), indicating that p53 was inhibited in its function in these cells. Cells harboring integrated HPV16 E6SAT genomes, when treated with actinomycin D displayed a heightened G1/S ratio indicative of p53 being functional (Figure 3D). One clone of NIKS harboring the HPV16 E6 SAT genome showed a heightened G1/S ratio (clone C), so we also analyzed the ability of clone D and E to attenuate or increase the actinomycin D induced G1/S ratio. The average actinomycin D induced G1/S ratio is shown in Figure 3E and demonstrates that clones harboring HPV16 E6SAT genomes have on average a greater magnitude in change in the G1/S ratio after actinomycin D treatment compared to NIKS not harboring HPV16 and that there is a large amount of variability in this change. Thus, the ability of E6 mutant genomes to be stably maintained as extrachromosomal, nuclear plasmids does correlate with the ability of these E6 mutants to inactivate p53-dependent function.

### Expression of dominant negative p53 inactivates p53 in NIKS

**Figure 4:**
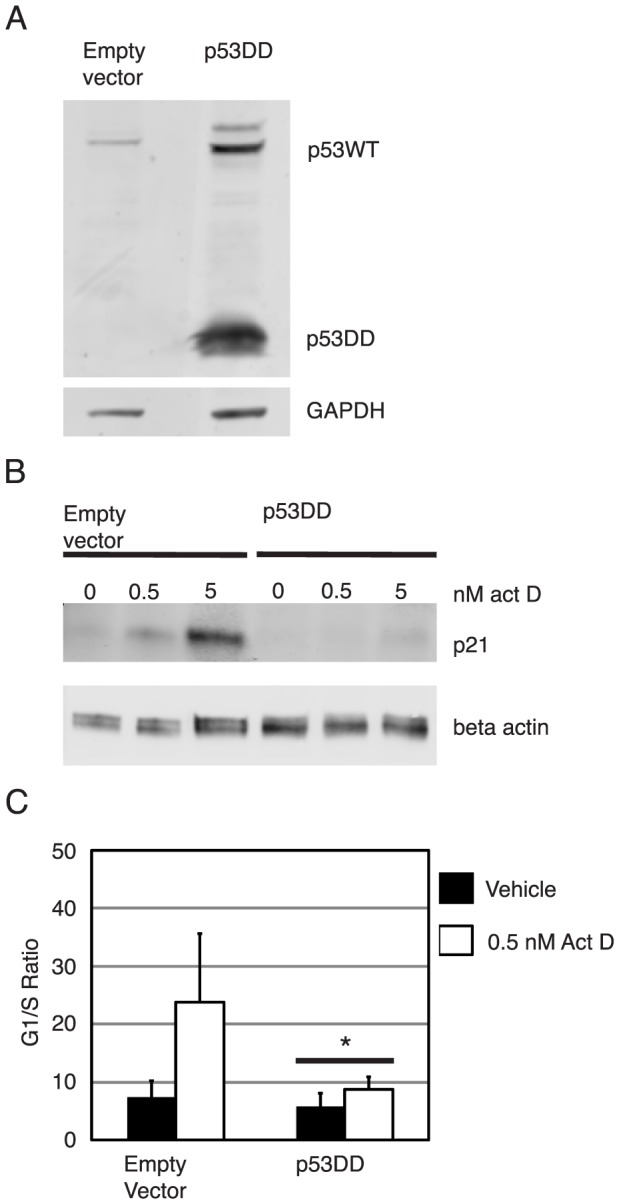
NIKS transduced with dominant negative p53 (p53DD), but not empty vector inactivate p53. Shown in panel (A) is a p53-specific western blot analysis of NIKS transduced with empty retrovirus vector (pLXSN: “empty vector”) or p53DD expressing recombinant retrovirus (pLXSN:p53, “p53DD”) using the pAB421 antibody. p53DD transduced NIKS, but not empty vector transduced NIKS express a low molecular weight p53DD protein. Shown in panel (B) is a p21-specific western blot of empty vector and p53DD transduced NIKS that were treated with vehicle, 0.5 nM or 5 nM actinomycin D for 24 hours. Empty vector but not p53DD transduced NIKS had an actinomycin D dose dependent response of increasing p21 levels. Beta actin was used as a loading control. Shown in panel (C) is the G1/S ratio (percentage of cells in G1/percentage of cells in S – based upon flow cytometric analysis of PI-stained cells - see methods) of empty vector and p53DD transduced NIKS treated with vehicle or 0.5 nM actinomycin D for 24 hours. The Sen-Adichie test for parallelism was used to compare the magnitude of change in the G1/S ratio between vehicle and actinomycin D treatment for each cell line. Compared to empty vector NIKS, p53DD NIKS were attenuated in actinomycin D-induced G1/S growth arrest. An asterisk indicates p values less than 0.0001.

In order to determine if 16E6's inactivation of p53 is sufficient to account for E6's role in plasmid maintenance, we created clones of NIKS transduced with a dominant negative, deletion mutant form of p53 that encodes only amino acids 1–14 and 302–390 of the mouse p53 protein (p53DD) [60], [61]. The p53DD protein oligomerizes with p53WT and inhibits binding of p53WT to p53 specific DNA sequences [60]. This results in inactivation of p53WT as p53WT is consequently unable to transactivate p53 reporter plasmids and natural p53 target genes including *p21* and *HDM2* [60], [62], [63]. Clones of NIKS transduced with the empty retrovirus vector (LXSN) or retrovirus expressing pLXSNp53DD were created and total cell lysates were analyzed by western blot to determine the presence of p53DD. As seen in Figure 4A, the low molecular weight form of p53 is seen in p53DD transduced NIKS but not in the cells infected with the vector only. Consistent with previous reports, p53DD transduced NIKS also had increased steady state levels of p53WT [60].

Functional studies were then performed to confirm that p53DD inhibited p53WT in NIKS. We first analyzed the steady state levels of a p53 target gene, p21, to see if p53DD could attenuate p53-dependent transactivation. After plating NIKS and treating cells for 24 hours with vehicle, 0.5 nM actinomycin D or 5 nM actinomycin D, total cell lysates were harvested and steady state levels of p21 were analyzed by western blot. The steady state levels of p21 increased in an actinomycin D dose dependent response in empty vector transduced NIKS, but not p53DD transduced NIKS (Figure 4B). We also tested if p53DD NIKS could attenuate an actinomycin D induced, p53 dependent, G1/S growth arrest by treating cells with vehicle or 0.5 nM actinomycin D and analyzing the percentage of cells in G1 and S phase of the cell cycle by flow cytometry as described in the methods. The Sen-Adichie test for parallelism was used to compare the magnitude of change in G1/S ratio after vehicle or actinomycin D treatment in empty vector and p53DD NIKS. As shown in Figure 4C, p53DD NIKS significantly attenuated the actinomycin D induced growth arrest compared to empty vector transduced NIKS (p<0.0001). Together these results demonstrate that the presence of p53DD functionally inactivates p53WT in NIKS.

### Inactivation of p53 is necessary for maintenance of HPV16 in the absence of E6

**Figure 5:**
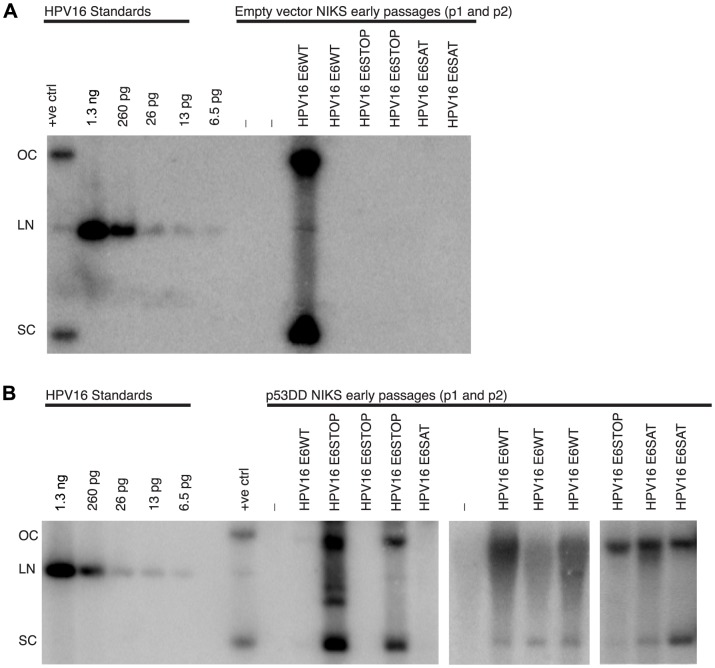
Inactivation of p53 is necessary for HPV16 genomes to be maintained. Shown are representative HPV16-specific Southern analyses of low molecular weight DNA from early passages of empty vector NIKS (A) or p53DD NIKS (B) that had been transfected with indicated wild type or E6 mutant HPV16 genomes (the symbol “-” indicates cells that were not transfected with HPV16 DNA). Low molecular weight DNA (3*10^6^ cells worth) was digested with XhoI and DpnI (non-cutters of mammalian replicated HPV16) prior to Southern analysis. Note the presence of circular wild type and E6 mutant HPV16 genomes in multiple populations of p53DD NIKS indicating that inactivation of p53DD rescues the plasmid maintenance defect of HPV16 E6STOP and HPV16 E6SAT genomes seen in parental NIKS.

We next asked if cells expressing p53DD could rescue maintenance of HPV16 E6 mutant genomes that were deficient for stable maintenance in parental NIKS. NIKS transduced with p53DD or NIKS infected with the vector only were transfected with wild type or E6 mutant (E6STOP, E6SAT) genomes and expanded populations were analyzed by Southern hybridization. While empty vector transduced NIKS supported maintenance of only the HPV16 E6WT genome, p53DD transduced NIKS supported maintenance of HPV16 E6WT, E6STOP and E6SAT mutant genomes (Table 2 and Figure 5). Thus, inactivation of p53 is necessary to account for E6's role in the maintenance of HPV16 as a nuclear plasmid.

**Table 2:** Frequency of NIKS:LXSN and NIKS:p53DD populations harboring extrachromosomal HPV16 DNA.

NIKS cell line	Transfected viral DNA	Fraction of populations analyzed that were positive for extrachromosomal HPV16 (%)
LXSN	none	0/8 (0%)
LXSN	HPV16 E6WT	3/8 (38%)
LXSN	HPV16 E6STOP	0/10 (0%)
LXSN	HPV16 E6SAT	0/10 (0%)
p53DD	none	0/7 (0%)
p53DD	HPV16 E6WT	9/11 (82%)
p53DD	HPV16 E6STOP	5/13 (38%)
p53DD	HPV16 E6SAT	6/12 (50%)

### Inactivation of p53 contributes to, but is not sufficient for stably maintaining HPV18 and 31 E6 null genomes at WT copy levels

**Figure 6:**
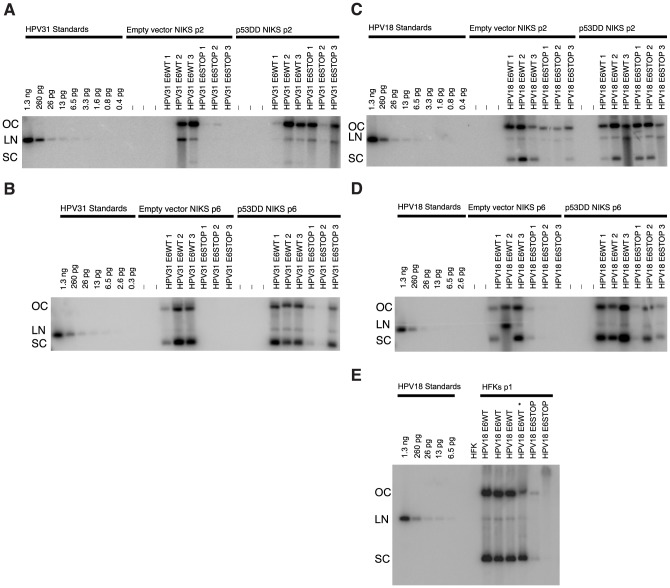
Inactivation of p53WT rescues maintenance of HPV31 and HPV18 E6 deficient genomes. Shown are HPV-specific Southern analyses of low molecular weight DNA (3*10^6^ cells worth, except for the lane in panel (E) marked with an asterisk which was only 1.7*10^6^ cells worth) from empty vector or p53DD NIKS that had been transfected with indicated genomes. DNA samples were digested with XhoI and DpnI (non-cutters of mammalian replicated HPV31 and HPV18) prior to Southern analysis. Three populations of transfected cells for each cell type were analyzed for each DNA construct. (A and C) Shown in these panels are DNA samples from the second passage of empty vector NIKS or p53DD NIKS transfected with either HPV31 (panel A) or HPV18 (panel C) genomes. (B and D) Shown in these panels are DNA samples from the sixth passage of empty vector NIKS or p53DD NIKS transfected with either HPV31 (panel B) or HPV18 (panel D) DNA genomes. HPV18 and HPV31 were excised from their respective bacterial vectors and used as standards; 1 copy of HPV/cell from 3*10^6^ cells is equal to 26 pg of HPV. Note that the order of populations harboring HPV genomes at passage 2 is the same order of the same populations harboring HPV genomes at passage 6. Panel (D) shows that as in NIKS, the HPV18 E6STOP genome was maintained at low copy numbers compared to HPV18 E6WT genomes in primary human foreskin keratinocytes (HFKs).

We were interested in determining if inactivation of p53 was sufficient to support maintenance of additional high risk HPVs, HPV18 and 31 in the absence of E6. To test this, empty vector and p53DD transduced NIKS were transfected with HPV31 E6WT, HPV31 E6STOP, HPV18 E6WT or HPV18 E6STOP genomes. In empty vector transduced NIKS, HPV31 E6STOP genomes replicated extrachromosomally at early passages (passage 1–2, passage 2 shown in Figure 6A), albeit at reduced copy numbers compared to HPV31WT genomes. Since some HPV31 E6STOP genomes were present in empty vector NIKS, we were interested in determining if these genomes were capable of stable maintenance. The HPV31 E6STOP genomes were not detectable at later passages of these same cell populations (passage 4–8; passage 6 shown in Figure 6B). In NIKS expressing p53DD, HPV31 E6STOP was maintained extrachromosomally at late passages but at a lower copy number compared to HPV31 E6 WT genomes in p53DD transduced NIKS (Figures 6B).

In empty vector transduced NIKS, HPV18 E6STOP genomes also replicated extrachromosomally at early passages (passage 1–2, passage 2 shown in Figure 6C) with copy numbers similar to that of HPV18 E6WT. At later passages (passage 4–8; passage 6 shown in Figure 5D), however, we again saw loss of HPV18 E6STOP replicons. In 2 of the 3 populations, there were barely detectable viral genomes present at passage 6, while the third population showed a reduced copy number compared to the populations harboring HPV18 WT. By passage 8 the latter population that retained low copies of HPV18 E6STOP at passage 6 had barely any detectable HPV18 signal (data not shown). In p53DD NIKS, all three populations that harbored HPV18 E6STOP at passage 2 retained it at passage 6, although there was a trend for the copy number of these genomes to decrease in comparison to HPV18 E6WT genomes in p53DD NIKS. Thus in the case for high risk HPV31 and 18 genomes, stable maintenance, as defined here as the retention of extrachromosomal replicons over at least 8 passages, depends heavily on the presence of the E6 oncogene, and this dependence can be partially rescued by inactivation of p53.

It is possible that the ability of HPV18 and 31 E6 null genomes to be maintained at low levels in early passages of NIKS is due to the immortalization characteristic of NIKS. To determine if this was the case, we co-transfected primary human foreskin keratinocytes (HFKs) with a drug resistance gene and either the HPV18 E6WT or E6STOP genome. Southern blot analysis of low molecular weight DNA from these cells, taken at 5.5 weeks post transfection when the cells had expanded sufficiently, demonstrated that HPV18 E6WT and E6STOP reproducibly replicated extrachromosomally in HFKs (Figure 6E). As observed in NIKS cells, the HPV18 E6STOP mutant genome replicated at a lower copy number compared to HFKs transfected with HPV18 E6WT mutant genome (Figure 6E).

## Discussion

Our studies demonstrate that HPV16 E6 is required for maintenance of the HPV16 genome as an extrachromosomal nuclear plasmid and that inactivation of p53 by dominant negative p53 (p53DD) is sufficient to support maintenance of the HPV16 genome in the absence of E6 (Figure 5). Inactivation of p53 was also necessary to support maintenance of HPV16 since the HPV16 E6SAT mutant genome, which is deficient for inactivating p53, was maintained only in NIKS transduced with p53DD (Table 2). Furthermore the ability of two other mutant genomes HPV16 E6I128T and HPV16 E6Δ146–151 to inactivate p53 (as shown by their ability to attenuate a p53-dependent G1/S growth arrest) correlated with the ability of these genomes to be maintained extrachromosomally (Figures 1B, 3C and 3D).

While inactivation of p53 was necessary for maintenance of HPV16 extrachromosomal genomes, decreased steady state levels of p53 were not necessary since the HPV16 E6I128T mutant is maintained extrachromosomally yet is deficient for decreasing p53 steady state levels (Figure 3A). While the E6I128T mutant is deficient for decreasing p53 steady state levels, this mutant is capable of preventing E7 mediated acetylation of p53WT at lysine 382 [56]. Acetylation of p53 at K382 increases the ability of p53 to bind DNA and the ability of E6 mutants to inhibit p53 K382 acetylation correlates with the ability of 16E6 mutants to resist interferon induced growth arrest [56], [64]. Thus, while the E6I28T mutant may not mediate p53 degradation, it may inhibit p53 function by preventing p53 acetylation. Cells harboring integrated HPV16 E6 SAT genomes had an increased magnitude of change in the G1/S ratio after actinomycin D treatment. There is no obvious explanation for this enhanced G1 arrest. It is possible that it reflects a consequence of this HPV genome being integrated in the host cell resulting in altered expression of cellular and/or other viral genes.

Our results also demonstrate that the PDZ binding domain of 16E6 is not required for stable maintenance of HPV16 because the HPV16 E6Δ146–151 (deletion of nucleotides 539–556) does not contain the PDZ binding domain yet is maintained extrachromosomally (Figure 1B). Others have demonstrated that an HPV16 E6 mutant with a stop codon introduced at amino acid 148, which truncates 16E6 at the PDZ binding domain, is deficient for stable maintenance [65]. Although both of these mutants lack the PDZ binding domain, the subtle differences in the specific mutations at the C terminus may differentially affect function and regulation of the 16E6 protein. As an example of how different C terminal mutations in E6 function, Kiyono et al. demonstrated that human mammary epithelial cells (HMECs) transduced with 16E6Δ140–151 fail to increase telomerase activity and fail to become immortalized while HMECs transduced with 16E6Δ146–151 can induce telomerase activity and can become immortalized [53]. It is possible that the 16E6Δ146–151 mutant removes a negative regulatory element of HPV16 E6, and this may account for the difference between our studies and the studies of Nicolaides et al [65].

Consistent with a previous report that HPV31 E6 is required for maintenance of HPV31 as extrachromosomal replicons [23], we detected only low levels of extrachromosomal HPV31 E6STOP genomes at early passages (passages 1–2), and these genomes were not detectable by passage 4. Inactivation of p53 rescued the ability of HPV31 E6STOP genomes to be stably maintained to passage 6 albeit at lower copy numbers than HPV 31 E6WT genomes (Figure 6B). In contrast, HPV18 E6STOP was maintained extrachromosomally in empty vector NIKS both at early and late passages, albeit at greatly reduced efficiency at the later passages (i.e., only 1 of 3 populations of HPV18 E6STOP retained detectable extrachromosomal HPV18 in the empty vector NIKS at passage 6, compared to 3 out of 3 retaining it at passage 2). These results indicate that there may be less of a requirement for E6 in the maintenance of HPV18 replicons than in HPV16 and HPV31. HPV18 E6STOP genomes were also maintained extrachromosomally but at lower copy numbers compared to HPV18 in HFKS, demonstrating that these results are not specific to NIKS. Inactivation of p53 did increase the capacity of the HPV18 E6STOP to replicate extrachromosomally over time, as all 3 populations of HPV18 E6STOP transfected p53DD NIKS retained extrachromosomal HPV18 at passage 6 (Figure 6D). Thus, we conclude that E6's inactivation of p53 contributes to plasmid maintenance for all three HPV genotypes tested.

Our detection of replicated HPV18 E6STOP and HPV31 E6STOP in early passages of NIKS demonstrates that E6 is not absolutely required for the establishment of the HPV18 and HPV31 genome as an extrachromosomal replicon. This is not surprising as it has been previously shown that the papillomaviral E1 and E2 proteins are sufficient to drive replication of plasmids containing the origin of papillomavirus DNA replication [15]–[17]. This result is also consistent with those published by Wang et al., which demonstrate that HPV18 E6 is required for robust amplification of HPV18, but nonetheless some amplification is detected in organotypic rafts of HFKs harboring genomes deficient in full-length E6 [31]. It is impossible to determine the requirement of 16E6 in the establishment of HPV16 given the absence of replicated HPV16 E6STOP at early passages. That we could not detect HP16 E6STOP genomes in empty vector NIKS at early passages may simply reflect the difference in the efficiency of replication of these genotypes in NIKS. HPV16 E6WT routinely gives rise to lower copy numbers of replicated viral genomes in NIKS (approximately 1–10 copies/cell) when compared to HPV31 and HPV18 (>50 copies/cell) as observed through Southern blot analysis.

Interestingly, ectopic expression of p53 attenuates establishment replication of BPV-1, HPV-16 and HPV-18 origin of replication in cells also expressing the respective E1 and E2 genes but does not affect maintenance of at least the BPV-1 origin of replication [66]–[69]. We have observed that inactivation of p53 rescues maintenance of HPV18 and 31 E6 null genomes at early passages, but the copy number/stable maintenance of HPV18 and 31 E6 null genomes decreases with time. Thus, it is possible that inactivation of p53 by E6 alleviates the negative influence of p53 during establishment and early maintenance. This could explain why inactivation of p53 restores copy numbers of HPV18 and 31 E6 null genomes to copy numbers seen in WT genomes at early passages but not late passages. The trend of HPV18 and 31 E6 null genomes to decrease in copy number over time in NIKS expressing p53DD (Figure 6C and 6D) may indicate that there are other activities of E6 that contribute to its role in stable plasmid maintenance. Alternatively, it is possible that residual p53 activity in the NIKS expressing p53DD (Figure 3B) is detrimental to the continued maintenance of HPV18 and HPV31 E6 deficient genomes.

Inactivation of p53 not only restored maintenance of HPV16 in the absence of 16E6, but also increased the efficiency of maintenance of HPV16 E6WT genomes: the HPV16 E6WT genome was maintained in 38% of empty vector populations vs 82% of p53DD populations (Table 2). One interpretation of this result is that HPV16 E6 is less efficient than p53DD at inactivating p53 and that the retention of some functional p53WT in NIKS harboring HPV16 E6WT genomes is responsible for attenuating HPV16 replication. This is consistent with others' findings [66], [68]. However, because the HPV16 E6WT genome replicated in 82% of the p53DD populations, whereas the HPV16 E6STOP genome replicated in only 38% of the p53DD populations and the HPV16 E6SAT genome replicated in only 50% of the p53DD populations, we further raise the possibility that an additional function of E6, independent of p53 inactivation, contributes to maintenance of HPV16. We hypothesize that this additional function of 16E6 is independent of the ability of HPV16 E6 to increase telomerase activity because HPV16 E6SAT can increase telomerase activity [53], [54] but is not maintained as efficiently as HPV16 E6WT genomes in p53DD NIKS, and because NIKS are inherently immortalized independent of E6 [50]. One possible role of 16E6 that may contribute to maintenance of HPV16 is E6 mediated transcription from the HPV16 LCR [70], [71].

Inactivation of p53 may be important to prevent apoptosis or senescence induced by the presence of the HPV genome and consequently E6 may prevent the loss of cells stably retaining HPV genomes. HPV-induced apoptosis or senescence could be triggered by the induction of DNA damage responses (DDR). During establishment of HPV18, Reison et al. demonstrated that the HPV18 genome co-localizes with γH2AX, a marker of DDR, [72]–[75] and likewise during maintenance of HPV31, the HPV31 genome co-localizes with several DDR components including pATM (S1981), γ-H2AX, 53BP1, Brca1 and Chk2 [76]. The ability of high risk HPV genomes to activate the DDR can be extended to HPV16; Sakakibara et al. demonstrated that human foreskin keratinocytes (HFKs) harboring extrachromosomal HPV16, 18 and 31 genomes express higher amounts of phosphorylated Chk2 (T68) than normal HFKs [77]. Activation of ATM as a consequence of DNA damage can lead to subsequent phosphorylation of p53 and consequently growth arrest, senescence and apoptosis (reviewed in [78]). If the DNA damage response activated during the life cycle of HPV leads to activation of p53, HPV E6 may be needed to inactivate p53 and thereby allow for the survival of the host cell. While we have attempted to follow the fate of cells transfected with HPV16 E6WT, E6STOP and E6SAT genomes, low transfection efficiencies of NIKS hampered our ability to determine if cells transfected with HPV genomes defective for inactivating p53 underwent a higher rate of growth arrest or apoptosis at early times following transfection. Results by Lepik et al., however, show that while ectopic expression of p53 attenuates papillomavirus replication during establishment, p53 expression failed to induce detectable apoptosis or growth arrest [66].

Alternatively, but not necessarily exclusively, HPV E6 may need to inactivate of p53 in order to attenuate an interferon-mediated innate immune response that could inhibit stable maintenance of HPV. An interplay between p53 and the interferon response pathway has been previously linked to inhibition of Sendai virus and vesicular stomatitis virus replication and may have similar effects on HPV replication [79], [80]. Consistent with this prediction, loss of extrachromosomal HPV16 genomes is correlated with an increased transcription of interferon-inducible genes [81] and treatment with interferon causes the loss of extrachromosomal papillomaviral genomes [82]–[84]. Notably, both HPV16 E6 and p53DD can decrease transcription of several interferon-induced genes in keratinocytes [85]–[87]. Thus, another possible explanation for how p53DD rescues the maintenance of HPV16 E6STOP genomes and confers stable maintenance of HPV18 and 31 E6 STOP genomes is the ability of p53DD to attenuate interferon signaling. Whether HPV needs to inactivate p53 in order to abrogate DNA damage responses or to inhibit interferon responses may not be mutually exclusive, since double stranded breaks can enhance interferon signaling through p53-dependent mechanisms [79], [88], [89].

Future work will be required to determine the exact reason behind the requirement of p53 inactivation for maintenance of HPV16 and stable maintenance of HPV18 and 31 and to identify additional roles of E6 that may contribute to maintenance of these genomes. Since p53WT negatively impacts establishment, maintenance and amplification of HPV papillomavirus genomes, it will be of interest to determine if p53 inhibits these different stages of replication in similar or different ways. Regardless, our results raise the interesting concept that drugs that can reactivate p53 in HPV-infected cells should be effective at eliminating persistent high-risk HPV infections and thereby reduce the risk of HPV-associated cancers in infected patients.

## Supporting Information

Text S1 Text S1 provides further details of the methods and materials.
(DOC)
