# Supplementary material for: Inactivation of p53 Rescues the Maintenance of High Risk HPV DNA Genomes Deficient in Expression of E6
Source: PLoS Pathog. 2013 Oct 24;9(10):e1003717. doi: 10.1371/journal.ppat.1003717 (PMC3812038; doi:10.1371/journal.ppat.1003717)
Supplement: Text S1 — provides further details of the methods and materials. (DOC) [file ppat.1003717.s001.doc]

**Text S1**

**Plasmids** The sequences of plasmids pcDNA6 (Invitrogen), pEGFP-N1 (BD Biosciences), pLXSN (Clontech), pCL-10A1 (Imgenex), pGL2-Basic (Promega) are each as described by the respective manufacturer. The plasmid carrying the HPV16 genome is as previously described (pEFHPV-16W12E GenBank Accession number AF125673) . The plasmid carrying the HPV18 genome (gift from Carl Baker) has the same sequence as the archetype HPV18 genome (pave.niaid.nih.gov) except that nucleotide (nt.) 4072 is an A and results in a missense mutation from phenylalanine to histidine at amino acid 46 of E5. The HPV31 full-length clone (gift from Carl Baker) has the same nt. sequence as GenBank Accession number J04353 except nts. 7332-7341 within the LCR are deleted . pLXSNp53DD is as previously described . pcDNA6.GFP was created by digesting each of pcDNA6 and pEGFP-N1 with BamHI and NotI. The eGFP fragment of pEGFP-N1 was then inserted into the BamHI/NotI site of pcDNA6. Site directed mutagenesis of E6 genes within the context of HPV16, HPV18 or HPV31 plasmids was completed with the QuickChange II XL Site-Directed Mutagenesis Kit (Agilent Technologies) according to the manufacturer’s protocol. The predicted sequence of the entire mutant genome was confirmed by sequencing with the BigDye® Terminator v3.1 Cycle Sequencing Kit (Applied Biosystems) at the University of Wisconsin Biotechnology Center DNA Sequence Facility. The HPV16 E6 amino acid numbers defining the mutant 16E6 genes are based upon the first amino acid being the methionine encoded by nts. 104-106. The HPV16 E6*I and E6*II mutants express only E6*I or E6*II due to deletion of an intron between nts. 227-408 and nts. 227-525 respectively (see also papilloma virus genome database (pave.niaid.nih.gov)). The primers used for site directed mutagenesis are as follows:

HPV16 E6 STOP Forward (F) 5’-GCA ATG TTT CAG GAC CCA CAG TAG CGA CCC AGA AAG TTA CCA CAG-3’

HPV16 E6 STOP Reverse (R) 5’-CTG TGG TAA CTT TCT GGG TCG CTA CTG TGG GTC CTG AAA CAT TGC-3’

HPV16 E6*I F 5’-ACA GTT ACT GCG ACG TGA GGT GTA TTA ACT GTC AAA AGC-3’

HPV16 E6*I R 5’-GCT TTT GAC AGT TAA TAC ACC TCA CGT CGC AGT AAC TGT-3’

HPV16 E6*II F 5’-CAG TTA CTG CGA CGT GAG ATC ATC AAG AAC ACG TAG-3’

HPV16 E6*II R 5’-CTA CGT GTT CTT GAT GAT CTC ACG TCG CAG TAA CTG-3’

HPV16 E6 SAT F 5’- CAG GAC CCA CAG GAG TCA GCC ACA AAG TTA CCA CAG-3’

HPV16 E6 SAT R 5’- 5’-CTG TGG TAA CTT TGT GGC TGA CTC CTG TGG GTC CTG-3’

HPV16 E6 I128T F 5’- CAA AGA TTC CAT AAT ACA AGG GGT CGG-3’

HPV16 E6 I128T R 5’- CCA CCG ACC CCT TGT ATT ATG GAA TCT TTG-3’

HPV16 E6 ∆146-151 F 5’-CAT CAA GAA CAT AAT CAT GCA TGG AG-3’

HPV16 E6 ∆146-151 R 5’-CTC CAT GCA TCA TTA TGT TCT TGA TG-3’

HPV18 E6 STOP F 5’-CAA TAC TAT GGC GCG CTT TTA GGA TCC AAC ACG GCG ACC CTA C-3’

HPV18 E6 STOP R 5’-GTA GGG TCG CCG TGT TGG ATC CTA AAA GCG CGC CAT AGT ATT G-3’

HPV31 E6 STOP F 5’-CCA TGT TCA AAA ATC CTG CAT AAA GAC CTC GGA AAT TGC AT-3’

HPV31 E6 STOP R 5’-ATG CAA TTT CCG AGG TCT TTA TGC AGG ATT TTT GAA CAT GG-3’

**Cells** NIKS (Normal Immortal KeratinocyteS) contain wild-type p53 and Rb sequences and were passaged at a density of 2*10^5^ cells per 10 cm dish containing mitomycin C treated feeder cells and complete E media (1 part high glucose, L-glutamine, phenol red containing Dulbecco’s Modified Eagle Medium ( DMEM (Invitrogen)), 1 part L-glutamine, phenol red containing DMEM/F12 (Invitrogen) supplemented with 5% fetal bovine serum (FBS), 1% penicillin/streptomycin (P/S (100 units/mL penicillin (Invitrogen) and 100 μg/mL streptomycin (Invitrogen)), 400 ng/mL hydrocortisone, 5 μg/mL insulin, 24 μg/mL adenine, 10 ng/mL epidermal growth factor and 8.4 μg/mL cholera toxin). When NIKS were grown in the absence of feeders, they were grown in low Ca^2+^ complete E media (1 part L-glutamine, phenol red containing DMEM/F12 (Invitrogen), 1 part home-made Ca^2+^ free DMEM supplemented with 5% chelex-treated FBS, 400 ng/mL hydrocortisone, 5 μg/mL insulin, 24 μg/mL adenine, 10 ng/mL epidermal growth factor, 8.4 μg/mL cholera toxin, 1% P/S and 0.275 mM Ca^2+^). At the time of passaging NIKS, feeders were removed by washing cells twice with phosphate-buffered solution (PBS (Invitrogen)), incubating the cells at room temperature (RT) with 3 mL of 0.02% EDTA (ethylenediaminediacetic acid tetrasodium salt in PBS) for 10 minutes and then tapping the sides of the dish to loosen the feeders. Feeders were aspirated, the cells were washed twice with PBS, incubated with 3 mL of 0.05% Trypsin-EDTA (Invitrogen) at 37°C, 5% CO_2_ for 10 minutes, resuspended well and then resuspended in 6-9 mL of E media. J2-3T3 mouse fibroblast cells were grown in DMEM containing 10% bovine calf serum (BCS), 1% P/S and split 1:20 every 3-4 days. Feeder cells were created by treating a confluent 15 cm dish of J2-3T3 cells with 10 mL DMEM media containing 10% BCS, 1% P/S and 4 μg/mL mitomycin C (MMC from *Streptomyces caespitosus*, Sigma) for 2-3 hours at 37°C 5% CO_2_. These MMC treated J2-3T3 cells were then washed twice with PBS, trypsinized with 3 mL 0.05% Trypsin-EDTA (Invitrogen) and resuspended in 9 mL complete E media. 1mL of these resuspended MMC treated J2-3T3 cells were added to a 10 cm dish containing 9 mL complete E media or 150 μL of MMC treated J2-3T3 cells were added to a 6 well dish containing 2 mL complete E media. The 293FT cell line (Invitrogen) was maintained in high glucose, L-glutamine, phenol red containing DMEM (Invitrogen) supplemented with 10% FBS, 1% P/S. 293FT cells were grown to confluency and split at a 1:200 dilution on a weekly basis. Media on all cells was changed every 2-3 days for a total of 3 times per week

**Creation of LXSN and p53DD transduced NIKS** 2*10^6^ 293FT cells were plated on a 10 cm dish in DMEM 10% FBS 1% P/S media. 293FT cells were then mock transfected or transfected with 12 μg of the retrovirus packaging vector pCL-10A1 and 12 μg of pLXSNp53DD vector or empty pLXSN vector using Lipofectamine 2000™ (Invitrogen) according the manufacturer’s protocol (except that only 18 μL of Lipofectamine 2000™ was used per 10 cm dish). Cells were incubated with the transfection complex for 4 hours before the transfection complex was removed, washed twice with PBS and fresh media added. The media was changed again 24, 48 and 72 hours after transfection. All of the 293FT virion containing media at 48 and 72 hours post-transfection was collected and pooled together (20 mL total volume) before being filtered through a 0.45 μm HV PDF syringe filter (Millex) and stored at -80°C until being used to infect NIKS. One day prior to infecting NIKS, 5.5*10^5^ of passage 36 NIKS were plated on a 10 cm dish in the presence of feeders in complete E media. On the day of infecting NIKS with pLXSN empty vector or pLXSNp53DD, virion containing media made from 293FT cells was thawed on ice. The media on NIKS was changed to 4 mL of virion containing media and 6 mL of complete E media containing 13.33 μg/mL polybyrene (for a final concentration of 8 μg/mL polybyrene). The day after infection, selection began with complete E media containing 250 μg/mL G418 (Gibco) for two days followed by complete E media containing 125 μg/mL G418 for two days. After selection, infected NIKS were grown in E media without G418 for 2 weeks and serially diluted 10 fold in 6 well dishes (1:10 to 1:10^6^ dilutions) containing complete E media and feeders. Individual clones in 6 well dishes initially diluted 1:1,000, 1:10,000 and 1:1,000,000 were then expanded. Expression of p53DD was determined by western blot and p53 function was determined by actinomycin D treatment and flow cytometry. LXSN and p53DD NIKS subsequently transfected with HPV genomes were co-transfected with pcDNA6.GFP and selected for with blasticidin.

**Preparation of HPV genomes for transfection** 10-100 μg of HPV16, 18 or 31 genomes were excised from the bacterial vector overnight with BamHI, NcoI or EcoRI (New England Biolabs) respectively, then re-ligated overnight at 16°C at a concentration of 8 ng/μL DNA in the presence of 15 Units/μL T4 DNA ligase (New England Biolabs) and 1X T4 DNA ligase buffer (New England Biolabs). Re-ligated DNA was concentrated by centrifuging the sample at 8,000 relative centrifugal force (RCF), RT, for 2 minutes and eluting the DNA with 50-500 μL sterile ddH_2_O pre-heated to 55°C. Gel electrophoresis was performed after each step to confirm digestion, re-ligation and recovery of concentrated DNA. DNA concentrations were determined by gel electrophoresis (0.8% agarose 1xTAE gel) and a NanoDrop 2000 spectrophotometer (Thermo Scientific).

**Stable transfections of HPV genomes** NIKS (3*10^5^) were plated in 5 mL of low Ca^2+^ incomplete (lacking epidermal growth factor) E media and in the absence of 1% P/S on a 60 mm dish without feeders. For transfections using Lipofectamine 2000™ (Invitrogen), cells were transfected with 3 μg of re-ligated HPV and 1.2 μg of pEGFP-N1 to select for neomycin resistant cells or 1.2 μg of pcDNA6.GFP to select for blasticidin resistant cells. HPV negative controls were transfected with 1.2 μg of pEGFP-N1 or pcDNA6.GFP alone. The manufacturer’s transfection protocol was followed except that the volume of dilution medium was 2 x 1.5 mL and only 6 μL of Lipofectamine 2000™ was used for each 60 mm dish. Cells were incubated with the transfection complex for three hours, then the media was removed and cells were washed three times with PBS and then 5 mL of low Ca^2+^ complete E media was added. For transfections using Effectene® (Qiagen), NIKS (3*10^5^) were seeded one day prior to transfection in a 60 mm dish containing low Ca^2+^ incomplete E media containing 1% P/S and no feeder cells. Cells were transfected with 400 ng of re-ligated HPV and 100 ng of pcDNA6.GFP according to the manufacturer’s protocol and a 1:25 ratio of DNA to Effectene® reagent was used. Cells were incubated with the transfection complex for 24 hours at 37°C 5% CO_2_. One-day post-transfection (for both Lipofectamine 2000 and Effectene® transfections), cells were transferred to a 10 cm dish containing feeders. Selection began 48-72 hours post-transfection by growing cells in the presence of 250 μg/mL G418 for two days followed by two days in the presence of 125 μg/mL G418 or by growing cells in the presence of 7 μg/mL blasticidin for four days. 14-21 days post-transfection, colonies were pooled together to form one population. At this point the population was referred to as passage 0 and was then passaged to a new 10 cm dish containing feeders as passage 1. Cells were passaged on a weekly basis and were approximately 90% confluent at the time of passaging and harvesting DNA.

**Establishment of clones harboring HPV16 genomes** Previously frozen populations of HPV-negative NIKS or NIKS harboring HPV16 were thawed in a 37°C water bath and immediately plated on a 10cm dish containing complete E media and feeder cells. Media was changed the next day and every 2-3 days thereafter. When 1-10 colonies large colonies were detected, these colonies were isolated by removing the media, washing twice with PBS and aspirating excess PBS at the border of each colony. A different pipette was used to aspirate the border of each colony. 20 μL of 0.05% Trypsin EDTA (Invitrogen) was added to each colony for five minutes. Individual colonies were then placed in individual wells of a 6 well dish containing feeder cells. Once individual wells were 90% confluent, feeders were removed and all NIKS were further expanded on a 10 cm dish containing feeder cells and complete E media. When the 10 cm dish became 90% confluent, low molecular weight or total genomic DNA was harvested and analyzed by Southern blot to determine the status of the HPV genomes.

**Harvesting of total genomic and low molecular weight DNA** For collection of total genomic DNA from NIKS, feeders were removed and NIKS were incubated overnight in a 10 cm dish at 37°C, 5% CO_2_ with 3 mL of complete total genomic lysis buffer (150 mM NaCl, 100 mM EDTA, 20 mM Tris pH 8.0, 1% sodium dodecyl sulfate (SDS) and 17 μL of fresh 20 mg/mL proteinase K). The next day, DNA was extracted with 3 mL each of phenol, a 50:50 phenol:chloroform mixture and chloroform. After each extraction, samples were centrifuged at 3,200 RCF, RT, for 5 minutes and then precipitated with 7.5 mL of RT 95% ethanol. Visible high molecular weight threads of DNA were removed and resuspended in 500 μL TE pH 8.0 (10 mM Tris-Cl pH 8.0, 1 mM EDTA) at RT overnight. The low molecular weight DNA was precipitated at -20°C overnight and then centrifuged at 7400 RCF, 4°C for 30 minutes. The supernatant was decanted, the pellet was air dried for 30 minutes, resuspended with 300 μL TE pH 8.0 and then combined with the previously isolated high molecular weight DNA. The resuspended DNA was incubated with 10 μL of RNAse A (10 mg/mL) at 37°C for one hour prior to being extracted with 800 μL each of phenol, 50:50 phenol:chloroform mixture and chloroform. After each extraction, samples were centrifuged at 16,000 RCF, RT, for 5 minutes. DNA was sheared by passing samples through a 22 gauge needle 40 times. The DNA was then precipitated overnight at -20°C with 80 μL 3 M NaOAc and 2.5 mL ice cold 100% ethanol. DNA was recovered by centrifugation at 16,000 RCF, 4°C, for 5 minutes. Samples were decanted, air dried for 30 minutes and finally resuspended in 50-200 μL TE pH 8.0. DNA concentrations were determined using a DU 7400 spectrophotometer (Beckman Coulter). To collect low molecular weight DNA, feeders were removed from NIKS and the total cell number was determined with a hemocytometer. Cells were centrifuged at 200 RCF, RT, for 5 minutes, the supernatant was removed and cells were washed with 2 mL PBS then centrifuged at 200 RCF, RT, for 5 minutes and the supernatant was removed. The cell pellet was resuspended in 600 μL complete Hirt resuspension buffer (150 mM NaCl, 10 mM EDTA and 20 mM Tris pH 7.4 and 15 μL of fresh 20 mg/mL proteinase K). The suspension was spiked with pGL2-Basic plasmid at a concentration of 50 copies per cell (for example, 1.8 ng of pGL2-Basic was spiked into 6*10^6^ cells) and then 2.4 mL of Hirt DNA lysis buffer (1.25 M NaCl, 10 mM EDTA, 20 mM Tris pH 7.4 and 0.75% w/v SDS) was added. This cell lysate was incubated at 37°C for 3 hours and then transferred to a high speed centrifuge tube and incubated at 4°C overnight. The lysate was centrifuged at 56,000 RCF for 1 hour at 4°C and the supernatant (low molecular weight DNA) was transferred to a round bottom 14 ml tube containing 7.5 ml ice cold 100% ethanol. Samples were inverted to mix, incubated at -20°C overnight, centrifuged at 7400 RCF, 4°C, for 45 minutes, and the pellet was allowed to air dry for 30 minutes before being resuspended in 300 μl TE pH 8.0. The resuspended DNA was transferred to a microcentrifuge tube and incubated at 37°C for 1 hour with 10 μl of 10 mg/ml RNAseA and extracted with 300 μl each of phenol, a 50:50 phenol:chloroform mixture and chloroform. After each extraction, samples were centrifuged at 16,000 RCF for 5 minutes at room temperature. DNA was precipitated at -20°C overnight with 750 μl ice cold 100% ethanol and 30 μl 3M NaOAc. Samples were then centrifuged at 16,000 RCF, 4°C, for 30 minutes and the DNA pellet was washed with 1 ml of ice cold 70% ethanol. The DNA was pelleted again by centrifugation at 16,000 RCF, 4°C, for 5 minutes and allowed to air dry for 30 minutes before being resuspended with TE pH 8.0 to obtain a concentration of 3*10^6^ cells worth of DNA in 20 μl.

**Southern hybridization** 20 μg of total genomic DNA or 3*10^6^ cells worth of low molecular weight DNA was digested overnight and electrophoresed in a 1x TBE (90 mM Tris, 90 mM boric acid, 2.5 mM EDTA) 0.8% agarose gel (Seakem LE Agarose Lonza Allendale, NJ) at 1 volt/cm for 18-24 hours. DNA digestion and loading was confirmed by staining the agarose gel in 5 μg/mL ethidium bromide in 1x TBE. The gel was then depurinated in 0.25 M HCl for 30 minutes, denatured in two changes of denaturing solution (1.5 M NaCl, 0.5 M NaOH) for 15 minutes each and neutralized in two changes of neutralizing solution (1.5 M NaCl, 1M Tris pH 7.4) for 15 minutes each. DNA was transferred to a positively charged nylon membrane (Hybond N+ Amersham Pittsburgh, PA) using the upward capillary transfer method with 10x SSC (1.5 M NaCl, 150 mM sodium citrate) for 24 hours. The efficiency of transfer was visualized by staining the agarose gel in 5 μg/ml ethidium bromide in 1xTBE. The nylon membrane was briefly rinsed in ddH_2_0 and dried on 3 MM blotting paper (MidSci St. Louis, Mo) for one hour before cross-linking with the auto crosslink function of the UV Stratalinker 2400 (Stratagene). After pre-hybridization of the membrane for 15 minutes at 51°C with Church hybridization buffer (250 mM Na_2_HPO_4_, 1% BSA, 245 mM SDS and 5 mM EDTA pH 8.0), the appropriate radiolabeled HPV16, 18 or 31 probe was hybridized overnight at 51°C. The membrane was washed five times, five minutes each, with Church wash buffer (140 mM SDS, 80 mM Na_2_HPO_4_) at 47°C. The hybridized membrane was exposed to a storage phosphor screen (Amersham Pittsburgh, PA) for at least 3 hours before being scanned with the Typhoon 8610 (Amersham). Brightness and contrast were adjusted using ImageJ version 1.46r to assist with visualization of the printed image. Radiolabeled probes were created by using the Rediprime II DNA Labeling System (Amersham) labeling kit to incorporate dCTP [α-^32^P] 6000 Ci/mmol EasyTide (PerkinElmer) into the HPV16 pEF399 plasmid or by 5’ end labeling 10 pmoles of oligonucleotides specific for HPV16, 18 or 31 (synthesized by Integrated DNA Technologies) in the presence of T4 polynucleotide kinase (PNK (New England Biolabs)), 1X T4 PNK buffer (New England Biolabs), 5 mM DTT and 75 μCi of ATP [γ-^32^P] 6000 Ci/mmol EasyTide (PerkinElmer) at 37°C for 1-2 hours. Unincorporated nucleotides were removed with the micro bio-spin P-30 tris chromatography columns (Bio-Rad Hercules, CA) per the manufacturer’s protocol.

HPV16 Oligonucleotides:

1. 5’-aat cgg ttg aac cga aac cgg tt-3’
2. 5’-gca gac att tta tgc acc aaa aga gaa ct-3’
3. 5’-gga aca aca tta gaa cag caa tac aac aaa ccg-3’
4. 5’-gtg gac cgg tcg atg tat gtc ttg tt-3’
5. 5’-aag acc tgt taa tgg gca cac tag gaa tt-3’
6. 5’-aat gac agt gat aca ggt gaa gat ttg gta gat-3’
7. 5’-agt att tgg gta gtc cac tta gtg ata tta gtg gat g-3’
8. 5’-gac tga aac acc atg tag tca gta tag tgg tg-3’
9. 5’-gtg ttg cga ttg gtg tat tgc tgc att-3’
10. 5’-gtc tcc aat gtg tat gat gat aga gcc tcc-3’
11. 5’-ggc aga cac taa tag taa tgc aag tgc c-3’
12. 5’-gtg ata ggg tag atg atg gag gtg att gg-3’
13. 5’-ggt atc aag gtg tag agt tta tgt cat ttt taa ctg c-3’
14. 5’-ggt gca gct aac aca ggt aaa tca tta ttt gg-3’
15. 5’-gct ggt aca gat tct agg tgg cc-3’
16. 5’-aac tgg aaa tcc ttt ttc tca agg acg tgg-3’
17. 5’-gac tct ttg cca acg ttt aaa tgt gtg tca-3’
18. 5’-aag tat ggg tgt att ttt tgg tgg gtt agg aa-3’
19. 5’-gcc ctt ccg atc ctt cta tag ttt ctt tag t-3’
20. 5’-tcg cac aac aca aca agt taa agt tgt aga cc-3’

HPV18 Oligonucleotides:

1. 5’- GCG ACC CTA CAA GCT ACC TGA T -3’

2. 5’- ATA GCT GGG CAC TAT AGA GGC CA-3’

3. 5’- GAA ATT CCG GTT GAC CTT CTA TGT CAC G-3’

4. 5’-CAA CAT TTA CCA GCC CGA CGA G-3’

5. 5’-GAG CTA GTA GTA GAA AGC TCA GCA GAC G-3’

6. 5’- TGC ATC CCA GCA GTA AGC AAC AAT-3’

7. 5’-CAG ATG ACG AGG ACG AAA ATG CAA CA-3’

8. 5’-TAG AGA CAG CAC AGG CAT TGT TCC-3’

9. 5’-GGC TGG AGG TGG ATA CAG AGT TAA G-3’

10. 5’-ATG GCG GCA ATG TAT GTA GTG GC-3’

11. 5’-GCA ACA ACA GCA GTG TAG ACG GTA-3’

12. 5’-CCA CGT GTA CAG ATT GGG TTA CAG C-3’

13. 5’-GGG GAG TAT TAA TAT TAG CCC TGT TGC G-3’

14. 5’-CGT TGT TAC ACG TAC CTG AAA CTT GTA TGT TAA-3’

15. 5’-GCC TTA TTA GCA GAC AGC AAC AGC AAT-3’

16. 5’-CCA CAA TGT GCA AAC ATT ATA GGC GAG-3’

17. 5’-GGG AAC ATG GCA TAC AGA CAT TAA ACC AC-3’

18. 5’-CGG TTG CAG CAC GAA TGG C-3’

19. 5’-GCA AAT GGC CCT ACA AGG CC-3’

20. 5’-GGT CGG GAC CGA AAA CGG-3’

HPV31 Oligonucleotides:

1. 5’-GAA AGT GGT GAA CCG AAA ACG GTT GGT ATA-3’

2. 5’-TAA TGC AAA GGT CAG TTA ACA GAA ACA GAG-3’

3. 5’-GGT GTA TAA CGT GTC AAA GAC CGT TGT G-3’

4. 5’-GAA CCG GAC ACA TCC AAT TAC AAT ATC GTT-3’

5. 5’-GCA CAC AAG TAG ATA TTC GCA TAT TGC AAG-3’

6. 5’-GAC GGG ACA CAT AGT GAA CGA GAG A-3’

7. 5’-CTA GCA ATG GTA AAG CTG CTA TGT TAG GTA-3’

8. 5’-TTG GTA CAG AAC AGG AAT GTC AAA CAT TAG CG-3’

9. 5’-GTG GAA CAA TGT GTA GAC ATT ATA AAC GAG CA-3’

10. 5’-TTA GAT GAT GCT ACA ACG CCA TGT TGG-3’

11. 5’-GAC AGA TGG CCA TAC CTA CAT AGC AGA-3’

12. 5’-AAG TCT TGA ACT GTA TTT AAC TGC ACC TAC A-3’

13. 5’-ATA CCT AAC ACA GTA TCA GTG TCA ACA GGA-3’

14. 5’-ACT ATT TGT GTG TCT TGT CAT ACG TCC AC-3’

15. 5’-ACG TGC GTC TGC TAC ACA ATT ATA TCA AAC-3’

16. 5’-GAA GAA TCT GGA ATT GTT GAT GTT GGT GC-3’

17. 5’-GGG CGT CTG CAA CTA CTA CTT CTA C-3’

18. 5’-GCA CCC TAG TTA TTA TAT GTT AAA ACG TCG ACG-3’

19. 5’-GTT GGT TTA GAG GTA GGT CGC GG-3’

20. 5’-GGG GCA ATC AGT TAT TTG TTA CTG TGG-3’

**q-PCR**  To determine HPV16 copy number by q-PCR, 3 μg of total genomic DNA was spiked with 140 pg of DpnI sensitive pGL2-Basic plasmid and incubated overnight at 37°C in the presence or absence of DpnI. All samples were then treated with 10U of Exonuclease III (New England Biolabs) at 37°C for 30 minutes and then incubated at 80°C for 30 minutes. Cq value based standard curves were obtained by spiking seven 10 fold serial dilutions of pEFHPV-16W12E (40 ag - 40 pg or 0.0001-100 copies HPV16/cell) and 14 pg pGL2-Basic (50 copies/cell) in 300 ng of NIKS total genomic DNA. Each 20 μL singleplex PCR reaction was performed in triplicate for each DNA template using 1X SsoFast probes supermix with ROX (Bio-Rad), 765 nM HPV16 F primer (5’-CGT GCA TCG GCT ACC CAA CTT TAT-3’), 1.3 μM HPV16 R primer (5’-CCA CTA AAG AAA CTA TAG AAG GAT CGG AAG GG-3’) and 200 nM HPV16 probe (5’-/6-FAM/TAT TCC ATT /ZEN/ GGG AAC AAG GCC TCC CAC AGC TAC AGA TAC A/IABkFQ/-3’) or with 200 nM pGL2-Basic F (5’-CGC AGC CTA CCG TAG TGT TTG TTT-3’), 200 nM pGL2-Basic R (5’-TCC GGA ATG ATT TGA TTG CCA AAA ATA GGA TC-3’) and 200 nM pGL2 -Basic probe (5’-/HEX/CGA TGT ACA /ZEN/ CGT TCG TCA CAT CTC ATC TAC CTC CCG GTT T/IABkFQ-3’). Primer and probe sets were synthesized by Integrated DNA Technologies and each product spanned 3 native DpnI sites of pEFHPV-16W12E or pGL2-Basic. The CFX96 Real-Time PCR Detection System was used and programmed to 2 minutes 50°C, 10 minutes 95°C and 40 cycles each of 15 seconds 96°C and 1 minute 60°C. The copy number of HPV16 per cell type was determined using Cq values from DpnI digested samples and the Cq value based standard curve generated with the Bio-Rad CFX Manager 3.0 Software. The value derived from 2^((pGL2-Basic Cq digested)-(pGL2-Basic Cq undigested)) was used to confirm DpnI digestion for each sample and for all samples this value was greater than 100.

**p53 function assays** 2*10^6^ NIKS were plated on a 10 cm dish containing 10 mL of low Ca^2+^ complete E media (without P/S). 18 hours later the media was replaced with fresh media containing 0.5 nM – 5 nM actinomycin D (Sigma) or vehicle (DMSO). 24 hours after actinomycin D or vehicle treatment, the media was removed, cells were washed twice with PBS, trypsinized in 1 mL 0.05% trypsin-EDTA (Invitrogen) and finally resuspended in 3 mL media. Cells were harvested by centrifuging at 200 RCF, RT, for 5 minutes, washed once with PBS, centrifuged again, decanted and fixed by resuspending in 100 μL of ice cold 0.02% EDTA and 900 μL of ice cold 95% ethanol. Cells were gently swirled and stored at -20°C for at least 24 hours and up to two weeks before staining with propidium iodide (PI). To stain with PI, fixed samples were centrifuged at 300 RCF, RT, for 10 minutes, washed with 2 mL PBS and centrifuged again. The supernatant was decanted and the cells were resuspended in 500 μL PI staining solution (1 mg/mL RNAse A, 33 μg/mL propidium iodide, 0.1% triton X-100 and 0.05% bovine serum albumin in PBS) and stored overnight at 4°C. The next morning, cells were filtered through nylon mesh containing 50 micron pore openings (smallparts.com B001D8HVRI) and analyzed with a Becton Dickenson FACSCalibur and BD BioSciences CellQuest Pro Version 5.2.1 software. At least 20,000 single cells as determined by forward and side scatter were analyzed and single cells were gated using Flow Jo Version 9.4.11 software. Single cells were then determined to be in G1, S or G2/M based on the intensity of the PI staining and the percentage of cells in each of these cell phases was determined with Flow Jo software. The G1/S ratio was calculated by dividing the percentage of cells in G1 by the percentage of cells in S phase. The magnitude change in the G1/S ratio of control NIKS after vehicle and ActD treatment was compared to the magnitude change in the G1/S ratio of experimental NIKS after vehicle and ActD treatment using the Sen-Adichie test for parallelism in MSTAT version 5.5.1 software.

To determine the steady state levels of the p53 target gene, *p21/WAF1* (Entrez Gene ID: 1026) in LXSN or p53DD NIKS (4*10^5^) were plated in 6 well dishes with low Ca^2+^ incomplete E media (without P/S) in the absence of feeder cells. 48 hours later, the media was replaced with fresh media containing 0.5 nM - 5 nM ActD or vehicle (DMSO). 24 hours after drug treatment, media was removed, cells washed twice with PBS and cells were trypsinized with 0.05% trypsin-EDTA for 10 minutes at 37°C, 5% CO_2_. Cells were centrifuged at 16,000 RCF, 4°C, for 1 minute, washed with cold PBS, centrifuged at 16,000 RCF, 4°C, for 1 minute and the supernatant was aspirated. Pellets were resuspended in 200 μL of ice cold RIPA buffer (25 mM Tris pH7.6, 150 mM NaCl, 1% SDS, 1% IGEPAL and 0.1% sodium dodecyl sulfate freshly supplemented with 1x protease inhibitor cocktail (500 μM MOPS pH 8.0, 20 μM EDTA pH 8.0, 100 μM EGTA, 0.2% glycerol, 50 μM Tosyl-lysine chloromethyl ketone, 1 μM amino caproic acid, 10 μM benzamidine, 2 μM leupeptin, 2 μM pepstatin, 0.4 μM aprotinin, 0.0001% trypsin chymotrypsin inhibitor and 0.0004% sodium azide) and 1 mM PMSF) and 50 μL of 6x SDS loading buffer (300 mM Tris pH 6.8, 15% glycerol, 1.2% β-mercaptoethanol, 6% SDS, 0.012% bromophenol blue) was added. Samples were sonicated at 4°C with a Branson Digital Sonifier® 450 at 30% amplitude for 10 seconds with a break of 5 seconds after the first 5 seconds of sonication. Samples were boiled for 10 minutes, centrifuged for 10 minutes at 16,000 RCF, 4°C, and the supernatant was transferred to a new tube and stored at -80°C until analysis by immunoblot.

**Immunoblots** For detection of p53 steady state levels in NIKS harboring HPV16 genomes, feeders were removed when NIKS were 90% confluent. NIKS were then trypsinized with 0.05% trypsin-EDTA (Invitrogen), neutralized with two volumes complete E media, and centrifuged at 200 RCF, RT, for 5 minutes. Samples were then washed with 2 mL PBS and centrifuged at 200 RCF for 5 minutes at RT. The supernatant was removed and the cells were resuspended in 400 μL of ice cold complete HNTG buffer (HNTG stock buffer (50 mM HEPES pH 7.5, 150 mM NaCl, 0.1% Triton X-100, 10% glycerol) was made complete on day of use as 87.8% HNGT stock buffer, 1% triton X-100, 1 mM EGTA, 1x protease inhibitor cocktail (500 μM MOPS pH 8.0, 20 μM EDTA pH 8.0, 100 μM EGTA, 0.2% glycerol, 50 μM tosyl-lysine chloromethyl ketone, 1 μM amino caproic acid, 10 μM benzamidine, 2 μM leupeptin, 2 μM pepstatin, 0.4 μM aprotinin, 0.0001% trypsin chymotrypsin inhibitor and 0.0004% sodium azide) and 1 mM PMSF). Samples were rocked for 20 minutes at 4°C, centrifuged at 16,000 RCF, 4°C and the supernatant was transferred to a new microcentrifuge tube. Protein concentrations were determined using the Bio-Rad DC™ protein assay according to the manufacturer’s protocol and OD values were determined with a DU 7400 spectrophotometer (Beckman Coulter). 20 μg of cell lysates were further supplemented with dithiothreitol (DTT, to a final concentration of 100 mM DTT) and SDS loading buffer (to final concentrations of 50 mM Tris pH 6.8, 2.5% glycerol, 0.2% β-mercaptoethanol, 1% SDS, 0.002% bromophenol blue) and then boiled for 10 minutes before being analyzed. For analysis by immunoblot, cell lysates were separated on a 12% NuSep Longlife gel in running buffer (100 mM Tris, 100 mM HEPES and 0.1% SDS) at 90V for approximately 2 hours. Proteins were transferred to a nitrocellulose membrane (for detection of p53DD) or a PVDF membrane (for all other protein detection) for 40 minutes at 15V in freshly made transfer buffer pH 9.2 (480 mM Tris, 390 mM glycine, 10% methanol, 0.0075% SDS). During the transfer, the transfer box was surrounded by dry and wet ice. Following the transfer, membranes were blocked for one hour at RT or overnight at 4°C in PBST (1% Tween-20 in PBS for p53 and p53DD detection) with 5% non-fat dry milk or Sea Block (Thermo Scientific, for p21 detection) diluted with an equal volume of PBS. Subsequently, membranes were washed 3 times for 5 minutes each in PBST, incubated with primary antibody diluted in blocking buffer for two hours at RT or overnight at 4°C (for p53DD detection), washed with PBST 3 times for 5 minutes each at RT, incubated with secondary antibody diluted in blocking buffer for one hour at RT, washed 3 times for 5 minutes each at RT in PBST and proteins were detected with ECL plus (Amersham) according to manufacturer’s protocol (p53DD and p53 detection) or air dried (p21 detection of p21). Membranes were scanned with an Amersham Storm 840 imager (p53DD and p53 detection) or LI-COR Odyssey Imager (p21 detection) and image contrast and brightness were adjusted using ImageJ 1.46r software. Antibody sources and dilutions were as follows: mouse anti-p53 DO-1 (1:500 Santa Cruz Biotechnology sc-126 for detection of p53WT, Entrez Gene ID: 7157), mouse anti-p53 pAb421 (1:30 Calbiochem OP03T for detection of p53WT and p53DD), goat-anti mouse (1:5,000 Jackson Immunoresearch 115-035-174), mouse anti-GAPDH-peroxidase (1:5,000 Sigma G9295, Entrez Gene ID: 2597), mouse anti-p21 DCS-60 (1:500 Santa Cruz Biotechnology sc-56335, Entrez Gene ID: 1026), goat anti-mouse IR Dye 680 (1:10,000 Licor), rabbit anti-β-actin (1:5,000 Santa Cruz Biotechnologies sc-1616-R, Entrez Gene ID: 60) and goat anti-rabbit IR Dye 800 (1:10,000 Licor). Densitometry of the p53 and GAPDH bands was performed using ImageJ. Briefly, rectangles of equivalent sizes were used to measure the raw integrated density of p53 or GAPDH and background values in the same lane. Background values were subtracted and the resulting p53/GAPDH ratio was normalized to the control.

1. Flores ER, Allen-Hoffmann BL, Lee D, Sattler CA, Lambert PF (1999) Establishment of the human papillomavirus type 16 (HPV-16) life cycle in an immortalized human foreskin keratinocyte cell line. Virology 262: 344.

2. Goldsborough MD, DiSilvestre D, Temple GF, Lorincz AT (1989) Nucleotide sequence of human papillomavirus type 31: a cervical neoplasia-associated virus. Virology 171: 306-311.

3. Gottlieb E, Haffner R, von Ruden T, Wagner EF, Oren M (1994) Down-regulation of wild-type p53 activity interferes with apoptosis of IL-3-dependent hematopoietic cells following IL-3 withdrawal. The EMBO journal 13: 1368-1374.

4. Shaulian E, Zauberman A, Ginsberg D, Oren M (1992) Identification of a minimal transforming domain of p53: negative dominance through abrogation of sequence-specific DNA binding. Molecular and cellular biology 12: 5581.

5. Smotkin D, Prokoph H, Wettstein FO (1989) Oncogenic and nononcogenic human genital papillomaviruses generate the E7 mRNA by different mechanisms. The Journal of Virology 63: 1441.

6. Smotkin D, Wettstein FO (1986) Transcription of human papillomavirus type 16 early genes in a cervical cancer and a cancer-derived cell line and identification of the E7 protein. Proceedings of the National Academy of Sciences of the United States of America 83: 4680.

7. Allen-Hoffmann BL, Schlosser SJ, Ivarie CA, Sattler CA, Meisner LF, et al. (2000) Normal growth and differentiation in a spontaneously immortalized near-diploid human keratinocyte cell line, NIKS. The Journal of investigative dermatology 114: 444.

8. Lambert PF, Ozbun MA, Collins A, Holmgren S, Lee D, et al. (2005) Using an immortalized cell line to study the HPV life cycle in organotypic "raft" cultures. Methods in molecular medicine 119: 141-155.

9. Todaro GJ, Green H (1963) Quantitative studies of the growth of mouse embryo cells in culture and their development into established lines. The Journal of cell biology 17: 299-313.

10. el-Deiry WS, Tokino T, Velculescu VE, Levy DB, Parsons R, et al. (1993) WAF1, a potential mediator of p53 tumor suppression. Cell 75: 817-825.
